# Recent Progress in Improving the Performance of Infrared Photodetectors via Optical Field Manipulations

**DOI:** 10.3390/s22020677

**Published:** 2022-01-16

**Authors:** Jian Chen, Jiuxu Wang, Xin Li, Jin Chen, Feilong Yu, Jiale He, Jian Wang, Zengyue Zhao, Guanhai Li, Xiaoshuang Chen, Wei Lu

**Affiliations:** 1 State Key Laboratory of Infrared Physics, Shanghai Institute of Technical Physics, Chinese Academy of Sciences, 500 Yu-Tian Road, Shanghai 200083, China; chenjian975899@163.com (J.C.); wangjiuxu@mail.sitp.ac.cn (J.W.); lixin4@shanghaitech.edu.cn (X.L.); chenjin@mail.sitp.ac.cn (J.C.); yufeilong@mail.sitp.ac.cn (F.Y.); h503000740@126.com (J.H.); 52200920007@stu.ecnu.edu.cn (J.W.); zhaozy@shanghaitech.edu.cn (Z.Z.); xschen@mail.sitp.ac.cn (X.C.); luwei@mail.sitp.ac.cn (W.L.); 2 Hangzhou Institute for Advanced Study, University of Chinese Academy of Sciences, No. 1 Sub-Lane Xiangshan, Hangzhou 310024, China; 3 University of Chinese Academy of Sciences, No. 19A Yuquan Road, Beijing 100049, China; 4 School of Physical Science and Technology, ShanghaiTech University, Shanghai 201210, China; 5 Shanghai Research Center for Quantum Sciences, 99 Xiupu Road, Shanghai 201315, China

**Keywords:** infrared photodetector, optical field manipulations, metasurface integration

## Abstract

Benefiting from the inherent capacity for detecting longer wavelengths inaccessible to human eyes, infrared photodetectors have found numerous applications in both military and daily life, such as individual combat weapons, automatic driving sensors and night-vision devices. However, the imperfect material growth and incomplete device manufacturing impose an inevitable restriction on the further improvement of infrared photodetectors. The advent of artificial microstructures, especially metasurfaces, featuring with strong light field enhancement and multifunctional properties in manipulating the light–matter interactions on subwavelength scale, have promised great potential in overcoming the bottlenecks faced by conventional infrared detectors. Additionally, metasurfaces exhibit versatile and flexible integration with existing detection semiconductors. In this paper, we start with a review of conventionally bulky and recently emerging two-dimensional material-based infrared photodetectors, i.e., InGaAs, HgCdTe, graphene, transition metal dichalcogenides and black phosphorus devices. As to the challenges the detectors are facing, we further discuss the recent progress on the metasurfaces integrated on the photodetectors and demonstrate their role in improving device performance. All information provided in this paper aims to open a new way to boost high-performance infrared photodetectors.

## 1. Introduction

**Figure 1:**
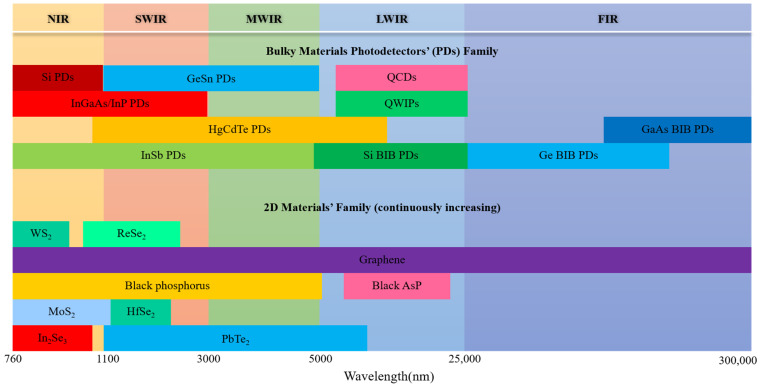
Some typical bulky photodetectors and 2D materials versus wavelength.

Photodetectors (PDs), the hub of photoelectric conversion, are the core of a series of devices that affect our daily lives. As one of the targets of photodetection, infrared (IR) radiation has attracted wide attention due to its capacity for working in harsh environments, with strong anti-interference and good concealment [1]. IR PDs’ applications in video imaging [2], optical communication [3], military radar [4], astronomical observation [5] and so forth have reached a high level of maturity due to the development of traditional materials and manufacturing technologies. The natural band gap of the material makes the detection of each band have a corresponding material, as shown in Figure 1. According to the different detection objects, it can be divided into five bands: near infrared (NIR), short-wave infrared (SWIR), mid-wave infrared (MWIR), long-wave infrared (LWIR) and far infrared (FIR). The most common detectors in several wave bands are silicon PDs for NIR [6], InGaAs and GeSn PDs for SWIR [7,8], HgCdTe PDs and InSb PDs for MWIR [9,10], and quantum-well infrared photodetectors (QWIPs), quantum cascade photodetectors (QCDs), as well as type II superlattice photodetectors for LWIR [11,12]. Note that the family of photodetectors in NIR to MWIR is very large, and a variety of alternative plans [13,14] for InGaAs and HgCdTe have been proposed. For FIR detection, it is often necessary to operate at low temperature due to the narrow bandgap of the PD material and Johnson thermal noise. More critically, the high doping concentration in the absorption layer gives rise to an inevitable conductivity effect in the impurity band, hence resulting in unacceptable dark currents and noise [15,16]. Thus, high-sensitivity FIR detection requires special detector designs, such as blocked impurity band (BIB) detector or quantum-well detector [17]. However, with decades of development, the performance of bulk materials photodetectors is reaching an upper limit. For some rigorous demands in terms of speed, efficiency or wavelength range, existing devices are not good enough. 

Two-dimensional (2D) materials are viewed as prospective candidates for the next generation of optoelectronic detection materials due to their atomically thin thickness, stacking with van der Waals force and lack of dangling bonds [18,19,20]. Currently, 2D materials have formed a huge family, including graphene [18] with zero band gap, PtSe_2_ [21], PtTe_2_ [22], black phosphorus (BP) [23] and its derivative with narrow band gap, transition metal dichalcogenides [24,25] (TMDs) with middle band gap and boron nitride (BN) [26] with large band gap. Due to the different band gaps of various 2D materials, a large number of PDs based on 2D materials have been proposed and demonstrated [18,27,28]. IR PDs based on 2D materials have exhibited ultrafast speed and ultrahigh sensitivity in photodetection [20,29,30]. However, due to the limitation of intrinsic small absorption coefficient and small size, 2D material-based photodetectors that have response to blackbody are rarely reported. Detectivities or photocurrent derived from the blackbody measurement are always considered as key characteristics before the real application of a new type of photodetectors. In this regard, there is still a long way to for 2D PDs, let alone considering the focal array. Furthermore, the current 2D materials are short of compatibility with CMOS technology and scalability for mass production. 

For traditional bulk materials of IR PD or 2D IR PD, there is a necessary demand for high performance. However, it is very difficult to further improve performance and reduce the size by optimizing the tape-out process. Due to the rapid development of micro–nano processing technology, people have already manipulated the light–matter interaction on the wavelength scale by artificially designing nanostructures [31,32,33,34]. This means that it is possible to monolithically integrate artificial microstructures to obtain multifunctional photodetectors with enhanced performance. As a representative artificial microstructure, metasurfaces have attracted tremendous attention from researchers owing to their strong light field manipulation capability within the subwavelength scale which can control photons with multiple degrees of freedom. For example, multifunctional metadevices have be realized through engineering the unique response of the metaatoms to steer the polarization state, phase, dispersion and topological charges. It provides an entirely new approach for developing high-performance metasurface-integrated photodetector devices.

In this review, we will discuss the current state-of-the-art PDs based on bulk mate-rials, 2D materials and hybrid configurations. First, the experimental realization and detection mechanism of various kinds of PDs are exhibited, and some issues in current PDs are summarized. Subsequently, we introduce the optical field manipulation mechanism of metasurfaces and discuss its potential in monolithic integration with existing detection semiconductors. Some works corresponding to the integration of optical field manipulations and photodetectors are discussed and reviewed. Finally, we propose some scenarios for integrating metasurfaces with IR PDs to meet the urgent demand for high-performance devices.

## 2. Infrared Photodetectors Based on Bulk Semiconductors

The IR PDs based on bulk semiconductors have been widely applied in many areas. Higher operation temperature, higher quantum efficiency, lower noise, and lower power consumption are the mainstream developing directions. High-quality materials and complete manufacturing processes are the key to achieving high-performance IR PDs. The adoption of liquid phase epitaxy (LPE) [35], metal-organic chemical vapor deposition (MOCVD) [36] and molecular beam epitaxy (MBE) [37] has decreased defect density in material growth. However, obstacles caused by the intrinsic properties of materials, such as Johnson thermal noise, leakage current introduced by defect, low absorptive efficiency due to the essential physics and so forth, still restrict the performance improvement of corresponding devices. In order to solve this, a series of unique and delicate configurations are proposed and demonstrated with outstanding characteristics [17,38]. In this section, we discuss some bulky material-based IR PDs for various wavebands. The most commonly used photodetectors in each band, including InGaAs NIR and SWIR PDs, HgCdTe MWIR and LWIR PDs, QWIPs and QCDs, and BIB FIR PDs, are all discussed in detail. The current issues and development prospective of PDs are also analyzed and forecasted.

### 2.1. In_x_Ga_1−x_As NIR and SWIR Photodetectors

**Figure 2:**
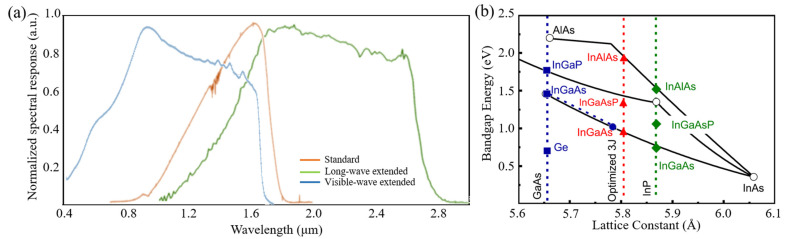
InGaAs PDs. (**a**) The normalized spectral response of standard, long-wave extended and visible extended InGaAs PDs. Date from [7,40,41]. (**b**) Bandgap as a function of lattice constant for III-V binary and ternary compounds. Reproduced with permission from Marina S. Leite et al., Applied Physics Letters; published by AIP, 2013 [42].

In_x_Ga_1−x_As, a ternary III-V semiconductor with tunable bandgap (0.35–0.77 eV) [39] resulting from tunable components of Ga and In, was widely applied in NIR and SWIR photodetection. According to the waveband of detection, PDs based on InGaAs are divided into two categories as follows: standard InGaAs PDs [40] and extended InGaAs PDs [7,41]. The corresponding response wavebands of standard, long-wave extended and visible extended devices are 0.9~1.7 μm, 0.9~2.8 μm and 0.4~1.7 μm, respectively, as shown in Figure 2a. For bulk materials, lattice matching is the basis of epitaxial growth of high-quality materials [42]. In_0.53_Ga_0.47_As, as the most representative material in this family of compounds, is perfectly lattice matched to InP (marked by a red star in Figure 2b), which results in the standard In_0.53_Ga_0.47_As/InP PDs [7,43]. The structure of standard InGaAs PD, composed of InP substrate, InP buffer layer, InGaAs absorption layer and InP cap layer, is illustrated in the inset in Table 1. Due to a more mature growth process, a completely lattice-matched substrate and a high operating temperature, many institutes, such as SITP, CAS [44], UTC Aerospace Systems [45], XenICs [46] and Sofradir [47], have achieved large-scale, high-performance standard InGaAs devices. All four institutes achieved 640 × 512 focal plane array (FPA), noting that SITP, CAS achieved 4000 × 128 FPA and 95% quantum efficiency, and UTC Aerospace Systems achieved high D* of 2.5 × 10^13^ Jones.

InGaAs devices have for capacity of extending the detection wavelength because of the tunable components. As shown in Figure 2b, the high Indium component will cause a large lattice mismatching, which gives rise to high dark current due to the existence of a large number of stacking faults. Thus, it needs a new buffer layer to relieve interlayer stress. InAlAs is viewed as an ideal material to solve this problem due to its wide bandgap and lattice matched with high Indium component InGaAs [42]. Y. Arslan et al. proposed a scenario [41], as shown in the inset in Table 1, which consists of an InP substrate, a thin InP buffer layer, an In_x_Al_1−x_As graded buffer layer, an In_0.83_Ga_0.17_As absorption layer and an In_0.83_Al_0.17_As cap layer. They achieved 3.7 uA/cm^2^ dark current density and 0.9–2.65 μm spectral response at 200 K under 0.25 mV reverse bias voltage, and obtained 10^10^ cmHz^1/2^W^−1^ peak detectivity at room temperature. SITP, CAS and UTC Aerospace Systems are also interested in long-wave extended InGaAs devices. UTC Aerospace Systems achieved a series of linear arrays, and the dark current was 10~100 nA and quantum efficiency was above 50%. SITP, CAS achieved a 1024 × 256 FPA with 10 nA·cm^−2^ dark current density and 5 × 10^11^ Jones detectivity. Compared with the long-wave expansion of InGaAs photodetectors, dual-color InGaAs detectors can simultaneously detect visible and infrared and have attracted the attention of researchers [48,49]. Standard detectors cannot recognize visible light, mainly due to the absorption of visible light by InP material. Thus, whether it is a front-illuminated or back-illuminated device, the thickness of InP needs to be controlled. For front-illuminated devices, the thickness of the InP cap layer should be reduced, while for a back-illuminated one, the InP substrate should be removed and the thickness of InP buffer layer should be reduced. Martin et al. proposed a back-illuminated structure to achieve a visible and infrared dual-color InGaAs/InP detector [7]. They introduced an InGaAs etch-stop layer to completely remove the InP substrate and design an ultra-thin InP contact layer to reduce the absorption for visible light by InP. According to this method, UTC Aerospace Systems and XenICs achieved 1280 × 1024 and 640 × 512 FPAs, respectively, and the devices had low dark current and high quantum efficiency, illustrated in Table 1.

### 2.2. Hg_x_Cd_1−x_Te MWIR and LWIR Photodetector

**Figure 3:**
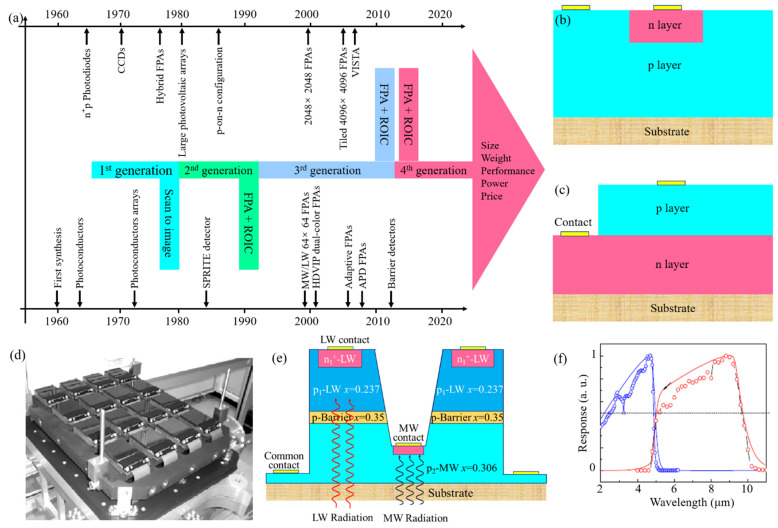
HgCdTe PDs. (**a**) Development of HgCdTe PDs. (**b**) HgCdTe n^+^p configuration. (**c**) HgCdTe p-on-n configuration. (**d**) Sixteen 2048 × 2048 HgCdTe arrays assembled for the VISTA telescope. Reproduced with permission from William Sutherland et al., Astronomy & Astrophysics; published by EDP Sciences, 2015 [57]. (**e**) Schematic of grooved HgCdTe two-color IR PD. (**f**) The spectral photoresponse of the LW/MW HgCdTe two-color IR PD with cut-off wavelengths of λ_1cut-off_ = 4.8 μm and λ_2cut-off_ = 9.7 μm at 0.01 V. Reproduced with permission from Weida Hu et al., Optics Letters; published by OSA, 2014 [64].

Similar to InGaAs, HgCdTe is a ternary II–VI semiconductor with tunable bandgap [50] (0–1.65 eV) due to the tunable component of Hg and Cd, whereas its detection bands are NIR to LWIR. However, NIR and SWIR HgCdTe PDs have a strict working temperature requirement, herein, we mainly discuss MWIR and LWIR HgCdTe PDs in this section. Since the materials were firstly synthesized in 1959, HgCdTe IR PDs have undergone four generations of development [51], as shown in Figure 3a. The first generation of HgCdTe PDs focused on single-element and hybrid array based on photoconductive and photovoltaic effects [52]. For the second generation, large arrays and new configurations were investigated to adapt to the demand of astronomical imaging and high operating temperature. The acronym of Signal PRocessing In The Element (SPRITE) detectors is the variation of standard photoconductive detector, which was utilized in a family of thermal imaging systems [53]. However, HgCdTe photoconductive devices were gradually abandoned in the late 1980s due to their large area array components requiring complex cooling methods. The structure of initial HgCdTe photovoltaic devices was an n^+^p junction, as shown in Figure 3b. In 1985, Rogalski A. and Larkowski W. proposed p-on-n configuration [38], shown in Figure 3c, and demonstrated its advantage of operating at high temperature. A series of configurations based on p-on-n configuration have been proposed and manufactured, such as double-layer heterojunction (DLHJ) [54] and double-layer planar heterostructure (DLPH) [55]. In the 1990s, the third generation of photodetectors focused on very large FPAs. Teledyne Imaging company developed 4096 × 4096 FPAs with low dark current (2.7 × 10^−13^ A) and high quantum efficiency (80%~90%) [56], shown in Figure 3d. Similarly, sixteen 2048 × 2048 FPAs [57], shown in Figure 3d, were loaded on the visible and infrared survey telescope for astronomy (VISTA).

The goal of the fourth generation of photodetectors is to achieve multicolor, multidimensional control and high-operating-temperature devices. In the early 2000s, multicolor devices attracted the attention of researchers due to the capacity of enhancing target discrimination and identification. The typical configuration of dual-color devices is shown in Figure 3e, in which the P layer is the barrier layer with a Cd mole fraction larger than that of the p_1_ and p_2_ layers. They grow a p_1_-P-p_2_ HgCdTe file on GaAs substrate by molecular beam epitaxy and etch the p_1_ and P layer through micro–mesa array technique until the p_2_ layer is exposed. Afterwards, the window of implantation is constructed by utilizing photoresist spray-coating technology. Figure 3f shows the spectral response of a back-illumination HgCdTe MW/LW dual-color with cut-off wavelengths of 4.8 μm for MW and 9.7 μm for LW. In addition to the p-on-n structure mentioned above, barrier structures were viewed as an effective strategy to achieve high-operating-temperature devices [58]. Its purpose is to adjust the valance and conduction band edges to block the transport of intrinsic carriers caused by temperature rise. Despite the offset in the valance or conduction band strictly limiting the performance, HgCdTe barrier PDs have a wide application in multispectral detection, and a fast response. To meet the high requirements of the fourth generation of photodetectors, an SWa3P (size, weight, performance, power and price) evaluation was proposed. For HgCdTe PDs, the best-performing substrate is CdTe, and the addition of a small amount of Zn can further improve its mechanical properties [59]. However, the growth of high-purity CdTe material is very difficult, thus, manufacturing large-area array devices based on this substrate still faces huge challenges. In order to solve this problem, devices based on Al_2_O_3_, GaAs and Si substrates have been proposed and investigated. A 2048 × 2048 FPA device based on Al_2_O_3_ and Si substrates has been fabricated [60,61]. The most common HgCdTe detector has a PIN structure, which can be divided into two types: planar type and mesa type. The mesa notch helps to reduce the crosstalk between the photosensitive elements [62], but also causes an increase in leakage current, so a more mature side passivation process is required. For this, CdTe film deposited by atomic layer deposition is the most potential passivation method due to its advantages of high resistivity, lattice matching and improved conformal coverage for high aspect ratio [63].

### 2.3. Quantum-Well Infrared Photodetectors, Quantum Cascade Photodetectors and Type II Superlattice Photodetectors

**Figure 4:**
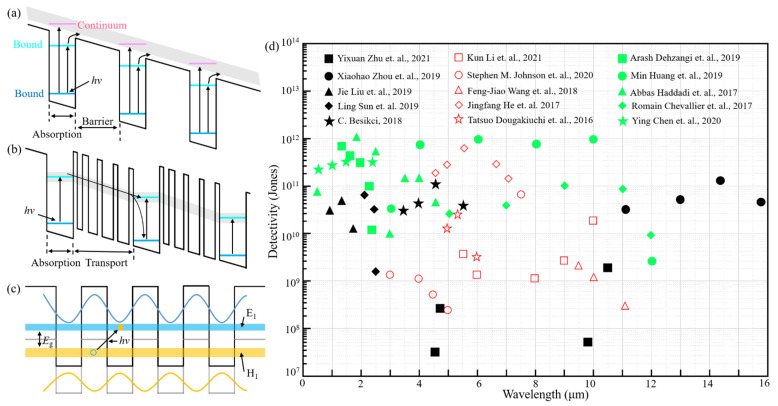
QWIPs and QCDs. (**a**) Carrier transport of QWIPs. (**b**) Carrier transport of QCDs. (**c**) Carrier transport of T2SL. (**d**) Detectivity as a function of wavelength for QWIPs (black solid point), QCDs (red hollow dot) and T2SL PDs (green solid point). Date from [65,69,70,73,74,75,76,77,78,79,80,81,82,83,84].

Compared with HgCdTe photodetectors, QWIPs have a more mature fabrication process and low costs [11], hence they develop rapidly. Unlike the conventional semiconductor detectors that generate electron-hole pairs based on the absorption of photons with energy larger than bandgap, QWIPs form the response based on the sub-band transition of carriers. As shown in Figure 4a, the well results from the high potential difference between the barrier layer and absorption layer. Under illumination, the carriers transition from the ground state to the first excited state, and the process of transition is divided into two situations. The first one is that the carriers transition from bound state to bound state [65], in this situation, the state of photoexcited carriers is still far lower than the top of the barrier, therefore, the photoexcited carriers need a large working bias to escape out of the well. By decreasing the thickness of well, the excited state could be elevated to the continuum state at the top of barrier [65]. The photoexcited carriers can directly transport into continuum, hence avoiding the assistance of high bias. For quantum cascade photodetectors, as shown in Figure 4b, the functional regions are the absorption layer and transport layer. The photoexcited carriers cascade to the next module ground state via a series of phonon ladders instead of continuum [11,65]. The auxiliary transport of phonon ladders results in high bias, which is not necessary for QCDs, thereby significantly reducing dark current and noise. The type II superlattice is formed by alternating and periodic arrangement of two lattice-matched semiconductors with sufficiently thin thickness [12,66], and its response waveband, theoretically covering 2~30 μm, varies with the thickness of semiconductor films. Similarly to QWIPs, there are electron wells and hole barriers in the two semiconductors, respectively. As shown in Figure 4c, the wave function of electrons in the well layer and holes in the barrier layer will overlap to form a continuous miniband, and the electrons and holes in these minibands will undergo transition with external assistance [67].

Although HgCdTe PDs develops rapidly, below 50 K, the advantage of HgCdTe PDs is less distinct due to the limitation of material growth. Due to that, QWIPs and QCD still have a certain range of applications. Figure 4c exhibits the detectivity as a function of wavelength for QWIPs and QCDs. For QWIPs, the detectivity of the proposed devices in each waveband can reach more than 10^10^ Jones, and even 10^11^ Jones. Besides, Yixuan Zhu designed MW/LW dual color QWIPs, and the detectivity can reach 1.8 × 10^9^ Jones at 10.4 μm and 2.6 × 10^8^ Jones at 5.8 μm. For QCDs, the detectivity of devices also can reach more than 10^10^ Jones at liquid nitrogen temperature, and can remain above 10^8^ Jones at room temperature. However, whether for QWIPs and QCDs, low quantum efficiency and low operating temperature are thorny issues. Although some solutions were proposed to enhance devices’ performance, the quantum efficiency is still below 40% [68]. Besides, there are some works on increasing the operating temperature to room temperature [69,70], but the detectivity is still below 10^9^ Jones. T2SL infrared photodetectors could alternate HgCdTe infrared photodetectors in the waveband range of LWIR [71,72,73]. The detectivity could even reach 10^12^ Jones at 8~10 μm [74]. Additionally, the detection waveband of T2SL SWIR PDs was extended to SWIR, and the detectivity can also reach 10^12^ Jones [75,76].

### 2.4. Blocked-Impurity-Band FIR Photodetector

**Figure 5:**
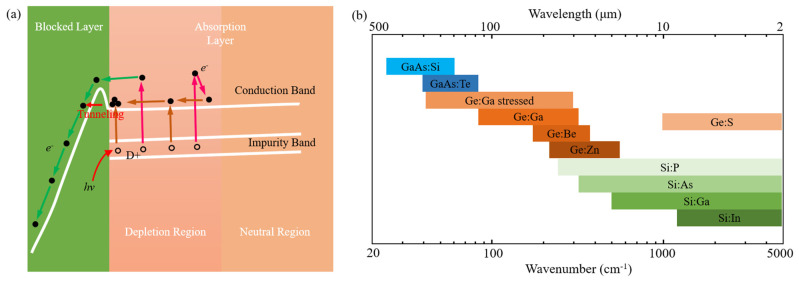
BIB PDs. (**a**) Photocarrier transport model of the BIB detector. (**b**) The detective range of various BIB detectors.

BIB IR photodetectors are developed on the basis of the extrinsic IR detector, and the entire extrinsic IR detector series can realize infrared detection in the wavelength range of 2~400 μm [15,85]. However, the extrinsic infrared detectors cannot meet the increasing requirements of infrared astronomical observation technology due to their nonlinear response and low resistance to cosmic radiation [16]. Consequently, in the design process of extrinsic infrared detectors, high doping concentration is required to achieve effective light absorption and high resistance is required to ensure that the electrons or holes bound at the impurity level are not ionized. In 1977, Petroff and Stapelbroek proposed a solution to achieve optically effective absorption and electrical high resistance by separating absorption layers and barrier layers, respectively, this designed structure is called BIB [17]. The energy band distribution in the doped absorption layer and intrinsic blocked layer are shown in Figure 5a. The wave function of the valence electrons of adjacent impurity atoms will overlap and the dispersed impurity energy level will form the impurity energy band as the doping concentration reaches a certain value [86]. However, the impurity band is blocked due to the low doping level in the blocked layer, thereby providing an obstacle for the dark current transits in the impurity band.

In the past 40 years, various BIB devices have been proposed and manufactured to detect information in far infrared. As shown in Figure 5b, the BIB devices are mainly divided into three types: Si-based, Ge-based and GaAs-based, which respond to cut-off wavelength of 40 μm, 240 μm and 400 μm. The different types of doping determine the response waveband of the devices, which is attributed to the different activation energy of the doping. Since the successful development of the first Si:As BIB device in 1980, BIB devices have attracted much attention from the scientists in developed countries. The Wide-field Infrared Survey Telescope (WISE) [87] and James Webb Space Telescope (JWST) [88] are equipped with the most advanced Si:As BIB FPAs, and the key performance is provided in Table 2. The development of Ge-based and GaAs-based BIB is very slow due to the severe material restriction and interdiffusion of impurities. As the core of far infrared detection, the development of BIB devices is significant. In recent years, some institutes from developing countries have also begun to manufacture related devices. Despite the lack of readout circuits working at extremely low temperatures, researchers can only manufacture unit devices, and they have obtained some interesting results. For example, Jin Chen et al. manufactured a mesa Si:P BIB device to eliminate the sharp response peak that is widely present in the spectra of planar structures through a tunneling current model [86]. Huizhen Wu et al. manufactured Ge:S BIB devices which respond to mid-infrared, and achieved a detectivity of 9.7 × 10^10^ Jones [89]. The corresponding performances are also illustrated in Table 2. Due to the response waveband being different, Si-based, Ge-based and GaAs-based BIB devices are not interchangeable. Currently, compared to the development of Si-based BIB devices, Ge-based and GaAs-based BIB devices are lagging. For Ge-based BIB devices, a small-scale array has been fabricated [90], but there is still a lot of space for development. The research on GaAs is even more behind, thus, the relative reports are very few [91].

## 3. Infrared Photodetectors Based on 2D Materials

Graphene, the most representative 2D material, exhibits various unprecedented properties, such as ultrahigh carrier mobility (10^6^ cm^2^V^−1^s^−1^) [27,95], quantum anomalous hall effect [96], excellent thermal conductivity [30], large theoretical specific surface area [29], and numerous applications from mechanics to space exploration. However, its applications in photoelectric devices are limited due to the zero bandgap. Researchers have also investigated other 2D materials with different bandgaps [97] and have endeavored to establish a new generation of photoelectric devices. BN [98] with large bandgap energy is viewed as the candidate that serves as an effective insulator and ultraviolet PD. BN can be used as a wrapping layer for other two-dimensional materials, thereby greatly improving the material mobility. TMDs [99] with middle bandgap energy are the most widely applied 2D materials. Monolayer and few-layer TMDs exhibit direct bandgap and indirect bandgap, respectively. With the increased layer thickness, bandgap energy decreases. The strong light–matter interaction between TMDs layers contributes to the formation of heterojunctions and homojunctions, with lattice match due to its atomic thickness. PtSe_2_ [21], PtTe_2_ [22], BP and its derivative [28], responding to MWIR, supplement the vacancy of narrow bandgap in the 2D materials family. Besides, BP with a polarization extinction ratio of approximately 7.8 dB [100] is a candidate in the field of polarized light detection. Moreover, the proposal and progress of various synthetic methods, such as mechanical cleavage [18], liquid exfoliation [101], ion intercalation and exfoliation [19] and chemical vapor deposition [102], etc., pushes forwards the rapid development of 2D PDs. The emergence of 2D materials brings new opportunities to break through the boundaries of the traditional semiconductor industry.

### 3.1. Graphene Bolometers

**Figure 6:**
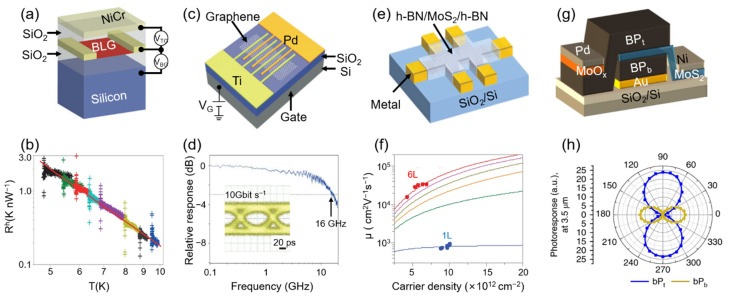
2D material-based photodetectors. (**a**) Device structure of a dual-gated bilayer graphene bolometer. Reproduced with permission from F. H. L. Koppens et al., Nature nanotechnology; published by Springer Nature, 2014 [26]. V_TG_, top gate bias; V_BG_, back gate bias. (**b**) Temperature dependence of heat resistance. Reproduced with permission from Jun Yan et al., Nature nanotechnology; published by Springer Nature, 2012 [103]. (**c**) Metal–graphene–metal (MGM) PDs with asymmetric metal contacts, and (**d**) Relative photoresponse versus light intensity modulation frequency. Reproduced with permission from Thomas Mueller et al., Nature Photonics; published by Springer Nature, 2010 [104]. Inset: receiver eye diagram obtained using this MGM photodetector, showing a completely open eye. Scale bar, 20 ps. (**e**) Schematic of the hBN-encapsulated MoS_2_ multiterminal device, and (**f**) The carrier density dependence of Hall mobility from 1 L and 6 L MoS_2_. Reproduced with permission from Xu Cui et al., Nature nanotechnology; published by Springer Nature, 2015 [105]. (**g**) Schematic of polarization-resolved BP/MoS_2_ heterojunction photodiode, and (**h**) Measured photoresponse under linearly polarized illumination at λ = 3 μm in BP_t_ and BP_b_ as a function of polarizer angle. Reproduced with permission from James Bullock et al., Nature Photonics; published by Springer Nature, 2018 [106].

Graphene can give rise to large light-induced changes in electron temperature due to its small electron capacity and weak electron–photon couple [27,95]. With this consideration, it is particularly suitable for bolometer devices that detect temperature-induced changes in electrical conductivity by light absorption. In [103], it was reported that two mechanisms resulted in bolometric photocurrents: bolometric effect dominates at low temperature and photoconductive effect dominates at high temperature. Figure 6a exhibits a dual-gated bilayer graphene bolometer with an optically transparent top gate [103]. The device was measured under illumination of continuous-wave CO_2_ laser light (10.6 μm) utilizing a four-probe signal by lock-in amplifier. The experimental heat resistance R^h^ depends on temperature T^−3.45^, as shown in Figure 6b, which is in consistence with the theoretical value. Furthermore, this kind of PD has low noise-equivalent power (33 fW Hz^−1/2^ at 5 K) and high intrinsic speed (>1 Ghz at 10 K), which are several times lower and five orders of magnitude higher than commercial silicon bolometers, respectively.

### 3.2. Metal–Graphene–Metal PDs

As the first reported 2D PDs, metal–graphene–metal (MGM) PDs are similar to traditional metal–semiconductor–metal (MSM) PDs [18,107]. The PDs were fabricated on a highly resistive silicon wafer with a thermal oxide. Single- or multi-layer graphenes were transformed and the electrodes were fabricated [108]. The photocurrent is mainly dominated by photovoltaic effect [109] and photothermoelectric effect [110], whereas the sign of photocurrent due to photovoltaic is opposite to that of the photothermoelectric effect [111]. Therefore, it is often used to distinguish the production mechanism of photocurrent. However, the low responsivity is always a serious problem. The built-in electric field only exists in a narrow region due to there being no blessing of external electric field [112]. As a result, carriers recombine but are unable to regenerate a considerable photocurrent carrier. The bias voltage solved the problem of insufficient photocurrent and caused an inevitable dark current at the same time. Thomas et al. solved this problem by using an asymmetric metallization scheme, as shown in Figure 6c [104]. They use palladium/gold and titanium/gold as the source electrode and drain electrode, and design a finger-crossing pattern to enhance the absorption of graphene. Figure 6d exhibits the relative photoresponse with a 3 dB bandwidth at 16 GHz. Additionally, they obtained a completely open eye at 10 Gbit s^−1^, demonstrating graphene’s capacity for error-free, optical data transmission.

### 3.3. Monolayer and Few-Layer MoS_2_ PDs

Due to its natural three-layer atomic structure [113], MoS_2_ has incomparable stability compared with graphene and is considered as the most promising 2D material. However, the measured mobility at room temperature is lower than the theoretically predicted value [20,114], which limits the application of MoS_2_ in optoelectronic devices. Using high-k dielectric materials to encapsulate MoS_2_ devices, the mobility increases to 15–60 cm^2^V^−1^s^−1^ from 0.1–55 cm^2^V^−1^s^−1^ [115]. The Schottky barrier formed by metal–MoS_2_ contact results in a considerable underestimate for actual values [24,116]. In addition to the purity of the material, dielectric environment and device structure strongly affect the electrical and optical properties [114]. Cui et al. proposed monolayer and few-layer MoS_2_ PDs [104] which were fully encapsulated by h-BN and electrically contacted by using gate-tunable graphene electrodes Figure 6e. As shown in Figure 6f, the mobility reaches 1020 cm^2^V^−1^s^−1^ for monolayer and 34,000 cm^2^V^−1^s^−1^ for six-layer MoS_2_ at low temperature, which enables the first observation of SDH oscillations in MoS_2_. Additionally, Zhang et al. achieved high detectivities of 10^13^ Jones and 10^15^ Jones in self-driven MoS_2_/Si heterojunction [117] and 3D RGO–MoS_2_/Pyramid Si heterojunction [118], respectively.

### 3.4. BP/MoS_2_ Heterojunction PDs

BP, an excellent 2D semiconductor that combines thickness-tunable bandgap and anisotropy [119,120], can cover the NIR and MWIR wavebands. Although it is easy to oxidize in the air, the antioxidation coating can effectively avoid this situation [121]. Furthermore, the combination of BP and TMDs have formed a series of functional van der Waals heterojunctions [23,25,122], which paves a new way for ultrathin IR PDs. However, compared to current IR PDs, the quantum efficiency of 2D material PDs is too low to characterize a wide-range continuous spectrum. Bullock et al. proposed a bias-selectable polarization-resolved BP PD with monolithic integration of two orthogonally aligned BP/MoS_2_ photodiodes, to measure both the intensity and polarization of MWIR [106]. Figure 6g exhibits the schematic of the two BP layers with perpendicular crystal orientations. They are separated by a common MoS_2_ electron contact and each BP layer has an isolated hole contact. The top BP layer contact is formed by MoO_x_/Pb, while the bottom one is formed by Au. Figure 6i shows the measured photoresponse as a function of polarizer angle under linearly polarized illumination at λ = 3.5 μm. The results demonstrate the different polarization selection of the BP_b_ layer and BP_t_ layer. Additionally, for both the bottom and top devices, the extinction ratio can reach about 100:1. The quantum efficiency and detection rate are 35% and 1.1 × 10^10^ Jones, respectively. Similarly, PdSe_2_ also has anisotropy. Jie et al. achieved an ultrahigh polarization sensitivity of 112.2 and high-resolution polarization imaging based on graphene/PdSe_2_/germanium [123].

## 4. Metasurface-Integrated Compact Photodetectors

Metamaterials are artificial electromagnetic materials composed of well-designed resonators, which are provided with novel optical properties and applications not accessible with natural materials [124,125,126,127,128]. For the purpose of flexible manufacture and integration, metasurfaces, a two-dimensional form of metamaterials, have been extensively developed to manipulate electromagnetic waves and to investigate the interaction of light with matter. In contrast to traditional optical components relying on the phase accumulation effect of light propagation, the design flexibility of the subwavelength resonators and their electromagnetic response allows the metasurfaces to achieve customized control of multiple free degrees of the light field, such as amplitude [129], phase [130,131], polarization [124,132], wavelength [133], and angular momentum [134,135]. Many novel optical effects and devices applications have been realized, such as utilizing the mode resonance to manipulate the amplitude to realize perfect absorbers [136,137] and amplitude-based holography [138]; engineering chromatic dispersion to realize the broad-band chromatic metalens [139,140,141], spectroscopy [142,143] and full-color imaging [144]; designing the birefringent meta-atoms for polarization conversion [125,132] and vectorial polarization holograms [145,146], etc. Additionally, with the development of optical devices integration requirements, the design of metasurfaces is not limited to manipulating single electromagnetic components. Developing towards multifunction design to deal with concurrent tasks is highly desired, which is further expanded by combining with novel materials and effects to provide infinite possibilities for the future of optical detections. Here, we will introduce the basic principles of metasurfaces and their integration with detectors from the aspects of enhanced absorption, polarization detection, broadband detection, and multidimensional detection in detail.

### 4.1. Enhanced Detector Absorption with Metasurfaces

**Figure 7:**
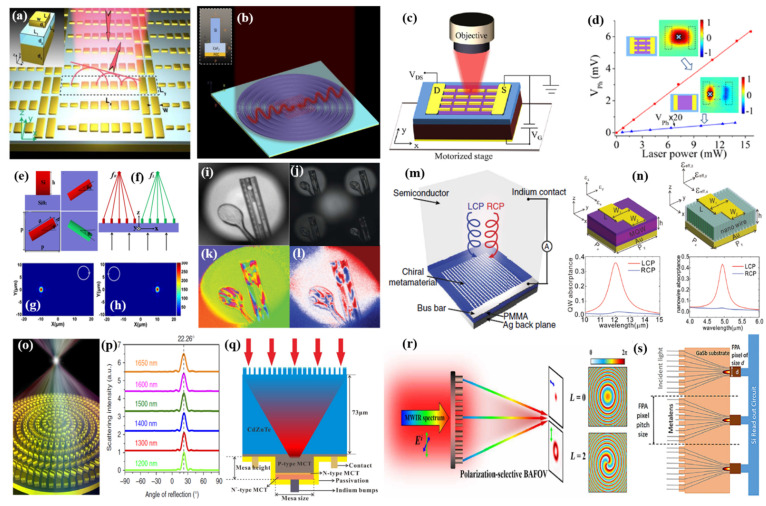
Metasurface-integrated infrared photodetectors. (**a**) Schematic of gradient metasurface. Reproduced with permission from Shulin Sun et al., Nano Letters; published by ACS, 2012 [147]. (**b**) Absorption enhancement of thin-layer black phosphorous with an all-dielectric gradient metasurface. Reproduced with permission from Nan Zhang et al., Optical Materials Express; published by OSA, 2021 [148]. (**c**,**d**) High-responsivity mid-infrared graphene detectors integrated with nano-antenna structures. Reproduced with permission from Yu Yao et al., Nano Letters; published by ACS, 2014 [150]. (**c**) A 3D schematic of photocurrent measurement setup. (**d**) Measured photovoltage of the graphene detectors with and without antennas as a function of incident laser power. (**e**–**h**) The broadband polarization-resolved devices based on the metalens in the near-infrared. Reproduced with permission from Hui Yang et al., Optics Express; published by OSA, 2018 [151]. (**e**) Schematics of designed meta-atoms. (**f**) Designed metalens with RCP and LCP lights are focused into two focal spots. (**g**) Simulated intensity profile under RCP incident light. (**h**) Simulated intensity profile under LCP incident light. (**i**–**l**) A plastic ruler and spoon are photographed with the polarization camera. Reproduced with permission from Noah A. Rubin et al., Science; published by AAAS, 2019 [132]. (**i**) A monochrome intensity image without polarization information. (**j**–**l**), respectively show a raw exposure, azimuth of the polarization ellipse, and the S3 component of the Stokes vector that describes circular polarization content. (**m**) Schematic of the CPL detector consisting of a chiral metamaterial integrated with a semiconductor that serves as a hot electron acceptor. Reproduced with permission from Li Wei et al., Nature Communications; published by Springer Nature, 2015 [152]. (**n**) The asymmetric metasurfaces integrate with quantum wells and InAsSb nanowire array to detect the CP light. Reproduced with permission from Xiaoshuang Chen et al., Advanced Optical Materials; published by Wiley, 2020 [153]. (**o**) Schematic for chromatic metalenses and (**p**) Simulated intensity of RCP-to-LCP scattering light vs. angle of reflection at various incident wavelengths. Reproduced with permission from Shuming Wang et al., Nature Communication; published by Springer Nature, 2017 [154]. (**q**) Schematic diagram of the metalens integrated HgCdTe infrared detector. Reproduced with permission from Fangzhe Li et al., Scientific Reports; published by Springer Nature, 2020 [155]. (**r**) Schematic illustration of the broadband achromatic focusing optical vortex generator with polarization-dependent functions. Reproduced with permission from Kai Ou et al., Science Advances; published by AAAS, 2020 [156]. (**s**) The imagination of a solid-immersion metalens integrated with an infrared focal plane-array pixel-unit cell. Reproduced with permission from Shuyan Zhang et al., Applied Physics Letters; published by AIP, 2018 [157].

As we described above, two-dimensional materials are ideal platforms for intriguing photodetection applications benefiting from their ultrathin thicknesses and excellent optoelectronic properties. However, being a thin layer, the intrinsically small optical absorption hinders its further development. Here, metasurfaces can not only increase the light absorption of infrared detection materials by carefully designing the subwavelength structures to transform incident optical waves into surface waves, but can also improve the light absorption capacity of the active area through metalens focus (the design principle is detailed in Section 4.2), without reducing the illumination area, while reducing the volume of the response material and suppressing dark current and thermal noise, which provides a solution to the problem of low absorption rate of thin-layer materials. Sun et al. [147] demonstrated a gradient metasurface by designing the geometry size of the antenna units to achieve 2π phase coverages, supporting high-efficiency (∼80%) anomalous reflections in a short near-infrared wavelength regime, as illustrated in Figure 7a. However, due to the high intrinsic ohimc loss of metallic structures and the lack of active tunability of permittivities at a specific wavelength range, the efficiency requirement of plasmonic metasurface-based devices is difficult to be fulfilled. Zhang et al. [148] elaborately designed the all-dielectric phase-gradient metasurface to convert incident light into surface wave, as shown in Figure 7b, which was integrated with BP to enhance the thin film absorption by 20 times in the mid-infrared. The Si-structure metasurfaces are compatible with existing semiconductor technologies, and are easy to integrate with multiple detection materials. It must be emphasized that, according to generalized Snell’s law [149], the normally incident light converting into a surface wave requires the phase gradient to be larger than the wave vector of the incident wave, and the incident angle to be larger than the critical angle. Furthermore, surface plasmon-enhanced photodetectors can not only achieve light trapping in the subwavelength range, but the enhanced local field distribution is conducive to the effective separation of electrons and holes to generate hot carriers. As long as the energy of the hot carriers is higher than the height of the Schottky barrier formed by the contact between the metal and the semiconductor, photodetection can be carried out, thereby breaking the limitation of the semiconductor band gap and broadening the response band of the photodetector. In a wide spectrum range, the specially designed metal nanostructure can excite surface plasmon resonance, and due to the abundant and diverse plasmon resonance modes it provides a variety of different design ideas for realizing photodetectors with excellent performance. Yao et al. [150] improved both light absorption and photocarrier collection in graphene detectors by integrating metal nanoantenna structures, as shown in Figure 6c,d. The metal antennas both concentrate free-space light to enhance the graphene light interaction and serve as electrodes to collect the generated photocarriers efficiently. As a result, the responsivity of metal antenna-assisted graphene detectors was enhanced more than 200 times at room temperature in the mid-infrared.

### 4.2. Metasurfaces Enabled Polarization Detection

To obtain the polarization state of light, the previous polarization measurement required the cascade of optical components such as waveplates, polarizers and detectors, which were difficult to integrate and to measure in real time. Metasurfaces provide an extraordinary and compact platform for manipulating polarization states of the light field. The polarization states’ manipulation with metasurfaces involves controlling wave-plate-like elements at the subwavelength scale, which imparts different phase shifts on incident polarized light along its fast and slow axes by rotating an angle relative to the reference coordinate system. There are several categories imparting polarization-dependent phase [125]: (1) The propagation phase can be imposed on each of two orthogonal, linear polarizations by adjusting element shape at each point of the metasurface. (2) The geometric phase, or Pancharatnam–Berry (PB) phase, can be imposed on two circular polarizations by rotating the orientation of the anisotropic element at each point. Specially, the PB phase increases linearly from 0 to 2π as the element is rotated at angles from 0 to π. (3) Combining the propagation phase and PB phase allows metasurfaces to impart fully independent phase profiles separately on any two orthogonal polarizations. We can design the meta-atoms through the above-mentioned phase-regulation mechanisms to achieve various productive functions. For example, metalens focusing is achieved by placing meta-atoms with different propagation phases in different positions to compensate the phase difference caused by propagation distance. The meta-polarimeter is realized by regarding the meta-atoms as waveplates and by controlling the geometric phase to perform polarization dectection. Furthermore, metagrating is utilized, based on the detour phase, through careful adjustment of the surface plasmon resonance gap mode. For the development of metasurface light field manipulations, it is inevitable that research will move in the direction of the independent degree of freedom parameters of the meta-atoms and combining multiple phase-control methods to simultaneously and independently control each dimension of the light field.

Yang et al. [151] demonstrated a polarization-resolved device composed two metalenses to focus two circularly polarized lights based on the PB phase, which can derive ellipticity based on the focusing efficiencies of two circularly polarized components, shown in Figure 7e,h. Moreover, the polarization-resolved device can work within a broadband wavelength range of 400 nm and be compatible with existing semiconductor technology, displaying potential applications in optical communications, sensing, and imaging. Noah et al. [132] presented a general polarization analysis method, matrix Fourier optics, to design and realize a compact full-Stokes polarization camera. Different from the past consideration of each element alone, this general method emphasizes the collective behavior of many elements at once, as expressed in the Fourier transform. This matrix Fourier method regards each optical element as a Jones matrix function. When the incident polarized field interacts with the optical elements, the angle spectrum response associated with polarization-dependent behavior can be obtained. In the article, they applied the polarization-dependent diffraction to the metagratings, analyzing arbitrary polarizations in parallel diffraction orders, and achieved full-Stokes polarization imaging without additional polarization assisting optical components, as shown in Figure 7i–l.

Circularly polarized light (CPL) detection is useful for enhancing security in free-space communication, remote sensing and infrared polarimetry imaging. However, the most optoelectronic materials are achiral and have no intrinsic circular polarization optical response. Recently, combining optoelectronic materials with chiral metasurfaces has provided a new way to enhance CPL detection. Li et al. [152] reported on a CPL detector integrated with hot electron-based chiral plasmonic metasurfaces to distinguish between left and right CPL without using additional polarization optical elements, shown in Figure 7m. The plasmonic elements also enable photon-energy harvesting though hot-carrier generation and injection, generating a novel photodetection and photocatalysis mechanism. Moreover, the working wavelength, bandwidth and polarization response of photodetectors are flexibly tunable by designing the plasmonic structures. Chu et al. [153] demonstrated that asymmetric, metamaterial-integrated quantum wells and InAsSb nanowire array exhibit a circular polarization extinction ratio of 14 and 12.6, respectively, in the mid-infrared regime, as shown in Figure 7n. Furthermore, Jiang et al. [158] designed a chiral plasmonic metasurface on the monolayer MoSe_2_ to achieve an ultrathin, compact, circular polarimeter, proving the universality and flexibility of the integration of two-dimensional optoelectronic materials with metamaterials.

### 4.3. Broadband Response Metalenses Integrated Detection

Chromatic aberration results from the variation in the lens focal length as a function of the incident wavelength, which greatly hinders full-color optical applications, such as in detection, displaying, imaging, etc. Traditional refractive optics eliminating chromatic aberration relies on cascading multiple lenses with complementary dispersion. This cascading configuration burdens optical systems in weight, complexity, and cost. Fortunately, broadband achromatic metalenses provide a new perspective on the development of miniaturized on-chip integrated optics, and portable, wearable devices, with the simultaneous independent adjustment of the phase and phase dispersion of the light field. The principle of being broadband achromatic is based on the fact that every nanostructure simultaneously fulfils the phase, group delay and group-delay-dispersion requirements. This condition can be comprehended as the nanostructures providing a larger time delay at the center of metalenses than on the sides, such that the transmitted wave packs leaving from different positions arrive and constructively interfere at the focus. Recently, various research teams have reported broadband achromatic metalenses, and initially established a general achromatic metaoptical theory. Wang et al. [154] demonstrated the reflective metal achromatic metalens working in the near-infrared band, as shown in Figure 7o,p, which was realized by introducing the secondary phase distribution profile at the reference wavelength with geometric phase and compensating the phase dispersion with the resonance phase to independently control phase and phase dispersion. However, the broadband achromatic metalenses utilizing the geometric phase and resonance phase is troubled by the problems of low focusing efficiency and polarization dependence on the incident light field. Fan et al. [141] overcomes the above issues of polarization dependence and low efficiency of achromatic metalenses by using symmetric meta-atoms. Ndao et al. [128] achieved polarization-insensitive, ultrawideband achromatic focusing at visible-near infrared wavelengths by numerical optimization algorithms, and the focusing efficiency exceeded 70%. Li et al. [155] reported on monolithically integrating a dielectric polarization-independent metalens into a HgCdTe infrared photodetector, as shown in Figure 7q. The metalens can enormously improve the absorptance, meanwhile reducing the photosensitive area and enhancing the detectivity. Compared with the devices without a metalens, the absorptance is kept above 50% (49 times higher) of the center wavelength of 4 μm. The photosensitive area shrinks from 40 μm × 40 μm to 5 μm × 5 μm, leading to the dark current reducing by 64 times, therefore, the detectivity increases 5.5 times. Moreover, the integration device exhibits broadband wavelength response in a range from 3.3 μm to 5 μm, with an average enhancement of detectivity by three times.

### 4.4. Mid-Infrared Multifunctional Compact Metadevices

Recently, multifunctional optical devices can manipulate multi-freedom of optical waves simultaneously and independently, which greatly expands the performance of optical devices and encourages more available applications. One significant development direction of multifunctional metadevices is to find new manipulation dimensions not limited to the abovementioned ones, and to use new mechanisms to improve device performance. Li et al. [159] introduced a general approach allowing the generation of vortex beams with different topological charges by the holographic metasurface, which makes it possible to take advantage of topology to realize robust optical transmission. Another intriguing development direction is the arbitrary and independent manipulation of multidimensions of optical waves. Since the different optical dimensions are associated with each other, finding the relationship between meta-atoms and dimensional terms to attribute each free degree to different paths becomes extremely difficult. One possible way is to control different parameters separately through spatial multiplexing technology, but this generally leads to efficiency degradation due to space-filling limitations and crosstalk between units. Zhou et al. [129] demonstrated bilayer metasurfaces to realize the manipulation of any two dimensions of optical waves, by combining different elements on each layer while maintaining high efficiency. On the other hand, bandwidth expansion is also extremely important for expanding the performance and functions of optical devices. Specially, as one of the atmospheric windows and molecular-vibration absorption bands, mid-wave infrared optics has many important applications, such as molecular fingerprint detection and free-space communications. Compared with visible and near-infrared, mid-wave infrared metadevices are rarely researched due to expensive experimental characterization equipment and scarce optical components. Ou et al. [156] realized the polarization-dispersion modulation, multifunctional metadevice in the mid-wave infrared range by using the all-silicon birefringent metasurface system, shown as Figure 7r. Within the 3.5~5 μm continuous bandwidth, different polarization-state photons will carry different orbital angular momentum information by metasurface device modulation, and will be focused on the specified focal plane with high polarization isolation. In addition, the adoption of all-silicon configuration can integrate with CMOS technology to realize integrated photonic devices, which will strikingly reduce the size and enhance the performance of devices due to compact multifunction manipulation. Zhang et al. [157] demonstrated a solid-immersion metalens etched on the backside of GaSb detector substrate material, to focus the broadband wavelengths (3.5~5 μm) on the infrared focal plane arrays, as shown in Figure 7s. The 10 × 10 metalens array shows an intensity enhancement of approximately 3 times, indicating improvement in the responsivity of the detectors. In terms of wide-field imaging, Xu et al. [160] proposed a silicon metalens array with polarization-multiplexed dual-phase design, to integrate with a COMS image sensor for wide-field microscopic imaging. This novel idea breaks through the limits of resolution and field of view, achieving wide-field imaging without reducing the resolution and guaranteeing unlimited space-bandwidth, which can inspire more revolutionary, compact, multifunction metadevices.

## 5. Conclusions and Outlook

In this review, infrared photodetectors and their integration with metasurfaces have been discussed. Researchers have indeed achieved exciting results on bulky material IR PDs in the past 70 years. There are devices with more than 1 million or even 10 million pixels in the 1~40 μm range, which significantly improves the capacity of humans to explore the world. According to the SWa3P standard, current bulky material PDs still have certain room for improvement in material growth and process optimization, but it requires more sophisticated instruments and more standard process flows. The trade-off of key properties, such as dark current and quantum efficiency, fix the limits of the devices. 2D materials were believed to break the performance limits, but their absorptivity and array integration are still being questioned. Furthermore, interesting avalanche effects of 2D materials have attracted much attention. Recently, the ballistic avalanche [161], unaffected by scattering, in a sub-mean free path in a 2D heterojunction, and avalanche effect in ultra-thin, depleted 2D film, were observed [162]. 2D materials still have great scientific research significance, and the feasibility of their application needs more urgent proof. Optimizing the structure or fabrication process to achieve a substantial increase in device performance seems to be a thing of the past, whereas integrating artificial microstructures with multidimensional control and enhancement is an effective method to realize the goal of the fourth generation of infrared photodetectors.

Absorptive enhancement is the first function applied to device integration. There are several strategies include converting incident light into surface waves and coupling into the semiconductor film and so on, thereby enhancing light absorption and significantly improving quantum efficiency. Due to this, the decrease in thickness of the active layer could reduce the overall dark current, and the absorptive enhancement could maintain the high quantum efficiency of devices. Multidimensional light manipulation from metasurfaces also provides a new prospective in multifunctional PDs, for example, traditional polarization detection always needs a complex design for components, which not only increases the complexity of devices but also weakens the optical signal and reduces the performance of devices. Metasurfaces with a capacity for polarization could be directly integrated on the substrate of devices, and greatly reduce the volume of the polarization system. In recent years, the integration of metalenses without chromatic aberration could achieve full-color optical detection, which indicates that people could enhance the collection of all information in the response waveband of PDs. Furthermore, the light manipulation of metasurfaces has been developed from a single-wavelength response to a broadband wavelength work, changed from one-dimensional manipulation to multidimensional joint manipulation, which would further contribute to the advancement of the fourth generation of infrared photodetectors.

Although the metasurface itself still has some problems, such as low efficiency, this does not hinder the dazzling applications. IR PDs, both traditional PDs and 2D PDs, with monolithically integrated metasurfaces, have great promise in photodetection. The on-chip integration of complex but multifunctional metasurfaces still has a long way to go; many metasurface-manufacturing processes for fragile substrates also need to be developed. Despite many difficulties, we firmly believe that the IR PDs with monolithically integrated metasurfaces, can break through the shackles of current PDs. Certainly, the application and manufacturability of IR PDs with monolithically integrated metasurfaces are still in the early or initial stages, but in the future, IR PDs with monolithically integrated metasurfaces will be an important part of next-generation IR detection systems.

## Figures and Tables

**Table 1:** A summary of InGaAs infrared photodetectors. Date from [44,45,46,47].

Structures	Institutes	AS, UTC	SITP, CAS	XenICs	Sofradir
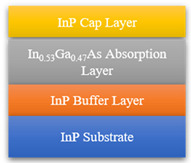 Standard	Arrays	2048 × 1 320 × 256 640 × 512	512 × 1 1024 × 1 320 × 256 640 × 512 4000 × 128	512 × 1 1024 × 1 2048 × 1 320 × 256 640 × 512	640 × 512
	DC or DCD	30 fA	<5 nA·cm^−2^	15~40 fA	30 fA
	D* or QE	2.5 × 10^13^ Jones	5 × 10^12^ Jones	80%	70%
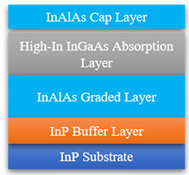 Long-wave Extended	Arrays	256 × 1 512 × 1 1024 × 1	640 × 1 512 × 256 1024 × 256		
	DC or DCD	10~100 nA	<10 nA·cm^−2^		
	D* or QE	>50%	5 × 10^11^ Jones		
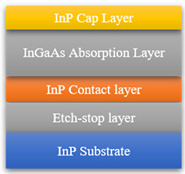 Visible Extended	Arrays	320 × 240 640 × 512 1280 × 1024		320 × 256 640 × 512	
	DC or DCD	<7.2 nA·cm^−2^		15~40 fA	
	D* or QE	2.9 × 10^13^ Jones		80%	

The insets are standard, long-wave extended and visible extended InGaAs infrared photodetectors, respectively. SITP, CAS: Shanghai Institute of Technical Physics, Chinese Academy of Sciences; AS, UTC: UTC Aerospace Systems; DC or DCD: dark current or dark current density; D* or QE: detectivity or quantum efficiency.

**Table 2:** A summary for BIB photodetectors. Data from [86,87,88,89,92,93,94].

Institutes	Detectors	Response Range	QE or D* or Responsivity	Dark Current	Array or Element
DRS (WISE)	Si:As	5~28 μm	>70%	10^−14^ A	1024 × 1024
Raytheon (JWST)	Si:As	5~28 μm	>70%	10^−14^ A	1024 × 1024
Zhejiang University	Si:P	~40 μm	1.6 × 10^11^ Jones	10^−9^ A	Element
	Ge:P	40~130 μm	1.3 × 10^13^ Jones	10^−9^ A	Element
	Ge:S	2~10 μm	9.7 × 10^10^ Jones	10^−9^ A	Element
SITP, CAS	Si:P	2.5~40 μm	5.6 × 10^12^~10^13^ Jones	10^−12^ A	Element
	Si:As	2.5~37 μm	5. 3 × 10^13^ Jones	10^−12^ A	Element
The 50th Research Institute, CETC	Si:P	7.4~46.3 μm	15.4 A/W	10^−12^~10^−9^ A	Element

CETC: China Electronics Technology Group Corporation.

## Data Availability

The data that support the findings of this study are available on request from the corresponding author on reasonable.
